# Analysis of online prescription patterns in Chinese patients with sequelae of cerebral infarction: a real-world study

**DOI:** 10.1038/s41598-024-62923-1

**Published:** 2024-05-25

**Authors:** Jia Tang, Tiantian Song, Ming Kuang, Hongying Liu

**Affiliations:** Hangzhou Kang Ming Information Technology Co., Ltd, 401 Building 4, Haichuang Park 998 Wenyi West Road, Yuhang District, Hangzhou, 310000 Zhejiang People’s Republic of China

**Keywords:** Sequelae of cerebral infarction, Prescription pattern, Real-world data, Chinese patent medicine, Western medicine, Neurological disorders, Neuroscience, Medical research, Neurology

## Abstract

Cerebral infarction (CI) is a common cerebrovascular disease worldwide, and the burden caused by the sequelae of CI has increased significantly. However, current treatment guidelines lack standardized recommendations for pharmacotherapy of sequelae of CI. This retrospective study collected and analyzed 1.98 million prescriptions concerning sequelae of CI from patients admitted to *Zhiyun Health Internet Hospital* in 2022. The mean age of patients was 66.2 ± 11.4 years, and 52.40% were male. 79.73% had one or more comorbidities. For treatment, the prescriptions of 1-, 2- and ≥ 3-drug accounted for 64.55%, 23.77% and 11.68% respectively. Chinese patent medicine (CPM) prescriptions, western medicine (WM) prescriptions, and CPM and WM combined (CPM + WM) prescriptions accounted for 53.81%, 27.33%, and 18.86% respectively. In CPM prescriptions, the most frequently prescribed medications were *Salvia miltiorrhiza* (34.81%), *Ginkgo biloba* (24.96%), *Panax notoginseng* (20.67%), *Gastrodia* (7.15%) and *Ligusticum Wallichii* (4.90%). For WM prescriptions, the most commonly prescribed agents were anti-hypertensive (32.82%), anti-thrombotic (16.06%), vasodilator (15.70%), anti-dementia (10.88%), and lipid-lowering (9.58%) drugs. Among CPM + WM prescriptions, 72.61% had CPM/WM = 1, 21.20% had CPM/WM < 1, and 6.19% had CPM/WM > 1. This research utilized real-world data extracted from internet hospitals in China to present valuable evidence of online prescription patterns among patients experiencing sequelae of CI.

## Introduction

The sequelae of cerebral infarction (CI), classified as I69.3 in the International Classification of Diseases and Related Health Problems, 10th Revision, fall under the broader category of I69, representing sequelae of cerebrovascular disease. "Sequelae of cerebral infarction", commonly known as "post-stroke sequelae", encompass a range of physiological, psychological, and functional impairments, as well as degenerative and disabling conditions, which occur with the onset and progression of stroke^1^.

According to the statistics from the World Stroke Organization, stroke is one of the leading causes of death and disability, particularly in developing countries where it has become one of the most prevalent cardiovascular diseases^2^. In China, more than 2 million new stroke cases are diagnosed annually, resulting in the highest number of Disability-Adjusted Life Years (DALYs) among all diseases^3^. As an age-related disease, the burden of stroke in China is expected to increase as the population ages. The direct cost of stroke in China among adults aged ≥ 45 years reached 1797.43 million US dollars in 2015, more than double the cost in 2011 (688.67 million US dollars)^4^. According to another study published in 2021, stroke imposes a significant economic burden, as estimated by the cost of illness method. The total economic burden ranges from approximately 1809.51 US dollars to 325,108.84 US dollars, with direct costs accounting for 86.2% and indirect costs accounting for 13.8%^5^.

Following acute treatment, post-stroke patients commonly experience various degrees of sequelae during the recovery period^6^. Common symptoms in post-stroke patients include pain, pseudobulbar affect, depression, anxiety, cognitive impairment, dementia, dysphagia, aphasia, epilepsy, gait instability, falls, and fractures^7,8^. These residual effects may lead to lasting impairments and limitations in daily activities, including weakness or paralysis on one side of the body, challenges with balance and coordination, and difficulties with language.

Both pharmacotherapy and physical therapy have demonstrated significant benefits as supportive care in alleviating symptoms for post-stroke sequelae. While physical therapy adheres to established standards of practice, such as those outlined by the American Physical Therapy Association, there is currently a lack of standardized practice guidelines for pharmacotherapy. Nonetheless, several medications have demonstrated clinical efficacy in addressing various manifestations of post-stroke sequelae, such as tizanidine, baclofen, dantrolene for limb dysfunction^9^ and memantine, donepezil for language dysfunction^10^. Chinese patent medicines (CPMs) are also used for neurological dysfunction^11^.

In this study, we used a comprehensive dataset of internet-based prescriptions in China to elucidate real-world medication choices and common prescription patterns by clinical practitioners for treating post-stroke sequelae. The primary objective is to provide substantial evidence for optimizing drug regimen of patients, and thereby improve their overall health outcomes.

## Methods

### Data source

This retrospective study utilized data from *Zhiyun Health Internet Hospital*. In accordance with Chinese laws and regulations, the data has been anonymized and structured to form a scientific research database, extracting essential information such as user IDs, age, gender, diagnosis (including primary and secondary diagnosis), prescription medications, dosage, and prescription date. The occurrence frequency of comorbidity was defined as one occurrence per prescription. All medications listed in online prescriptions were oral medications, which is in compliance with the "Measures for the Supervision and Administration of Online Sale of Medicinal Products" adopted by the State Administration for Market Regulation. We categorized all medicines into two groups: Chinese patent medicines (CPM) and western medicines (WM). CPMs were further categorized into CPMs with distinguishable main ingredients (MI-CPM) and CPMs without distinguishable main ingredients (NI-CPM), in accordance with the Pharmacopoeia of the People's Republic of China 2020. This study conducted a cross-sectional analysis of all prescriptions for the sequelae of CI between Jan 2022 and Dec 2022.

### Patients

The study focused on patients with sequelae of CI, who had received specific medication recommendations for the management of this condition. All participants in the study had previously been diagnosed with sequelae of CI in public or private primary healthcare facilities. Patients were eligible for inclusion if they were aged 18 years or older, had been diagnosed with sequelae of CI, had received a prescription for the first time on the online platform between Jan 2022 and Dec 2022, and had comorbidities associated with sequelae of CI (determined based on the diagnostic information provided in the prescriptions).

### Ethical consideration

Ethical assessment is not required prior to conducting the research reported in this paper, as the present study does not have experiments on human subjects and animals, and does not contain any sensitive and private information. The study used a database provided by *Zhiyun Health Internet Hospital*, and all data were encrypted to preserve patient privacy and confidentiality. This measure ensured that no individual's health data could be linked to specific individual. As per the Ethical Guidelines issued by the Office of the Medical Ethics Expert Committee of the National Health Care Commission of China, ethical approval and informed consent were not required for this study. All methods were carried out in accordance with relevant guidelines and regulations.

### Statistical analysis

Data was retrieved using structured query language, and variables such as patient diagnosis, gender, age, and medication categories were analyzed. The first online prescription for sequelae of CI between Jan 2022 and Dec 2022 was considered the index prescription, with the endpoint being the proportion of index prescriptions during the period. Prescriptions were categorized based on the presence of CPM and WM in the prescriptions. Mean ± standard deviation was used to present patient age, total number of medications, and other data. Categorical data, including gender, prescription patterns, and comorbidities, were presented as numbers and percentages. Python 3.7 was used for all statistical analysis.

## Results

### Clinical characteristics of cerebrovascular disease patients

Table 1 presents the clinical characteristics of patients with sequelae of CI. A total of 198,1450 valid prescriptions met the inclusion criteria for this retrospective study, and the average age of the patients was 66.2 ± 11.4 years. Among all the valid prescriptions, approximately 1.04 million were for male patients (52.40%). 20.27% of patients had no comorbidity, while 79.73% had at least one comorbidities. In all the patients, hypertension emerged as the most frequent comorbidity, occurring in 51.26% of the patients, followed by diabetes (29.04%), chronic kidney disease (27.38%), dyslipidemia (16.12%), and coronary artery disease (8.69%).

**Table 1:** Clinical characteristics of patients with sequelae of cerebral infarction (n = 1,981,450).

Characteristic	Overall population
Age (years)	66.2 ± 11.4
Male (n, %)	1,038,247, 52.40%
Number of comorbidites	
0	20.27%
1	60.34%
2	13.96%
≥ 3	5.43%
Proportion of each complication in all patients	
Hypertension	51.26%
Diabetes	29.04%
Chronic kidney disease	27.38%
Dyslipidaemia	16.12%
Coronary heart disease	8.69%

The gender and age distribution of the patients with sequelae of CI is shown in Table 2. In the total population, the youngest age was 18 years and the oldest was 96 years. Among them, a large majority of participants were > 65 years old, accounting for 61.58% of the patients.

**Table 2:** Distribution of patient’s gender and age.

Age (years)	18–65	> 65
Male (n, %)	408,042 (53.60%)	630,205 (51.65%)
Female (n, %)	353,231 (46.40%)	589,972 (48.35%)
All (n, %)	761,273 (38.42%)	1,220,177 (61.58%)

### Proportion of prescriptions

**Figure 1:**
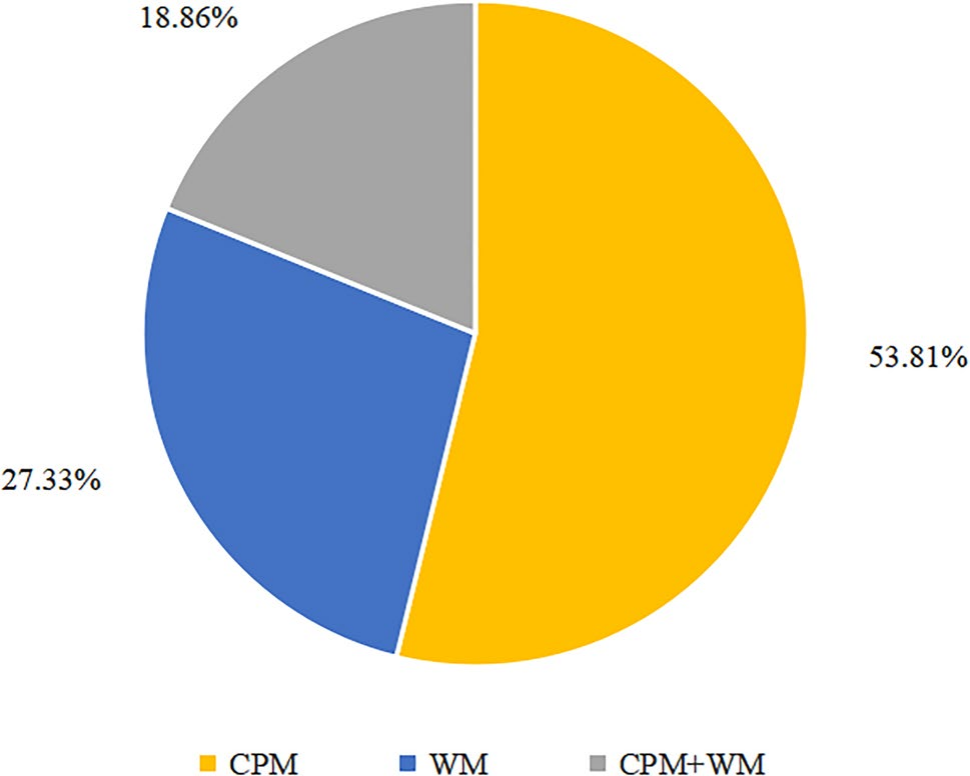
The proportion of prescriptions composed of CPM, WM, or CPM + WM in the overall prescriptions.

Among all the prescriptions, 53.81% contained only CPM, 27.33% contained only WM, and 18.86% contained both CPM and WM (Fig. 1). Table 3 shows the medication regimen for patients of different sexes and ages in detail. Among all prescriptions, CPM was the most common therapy among different age groups and sexes.

**Table 3:** Proportion of prescriptions in patients of different gender and age.

Variable	CPM	WM	CPM + WM
Sex			
Male	558,369 (53.78%)	283,857 (27.34%)	196,021 (18.88%)
Female	507,821 (53.84%)	257,683 (27.32%)	177,699 (18.84%)
Age (years)			
18–65	408,956 (53.72%)	208,969 (27.45%)	143,348 (18.83%)
> 65	657,309 (53.87%)	332,498 (27.25%)	230,370 (18.88%)

Of all the CPM prescriptions, MI-CPM accounted for 76.17%, while NI-CPM accounted for 23.83%. Among MI-CPMs, there is a high percentage of prescriptions containing the following traditional Chinese medicines (TCMs), including *Salvia miltiorrhiza* (34.81%), *Ginkgo biloba* (24.96%), *Panax notoginseng* (20.67%), *Gastrodia* (7.15%), *Ligusticum Wallichii* (4.90%), *Pueraria lobata* (0.44%), *Carthamus tinctorius* (0.43%), *Erigeron breviscapus* (0.29%), *Crataegus pinnatifida* (0.15%), and others (6.20%) (Table 4).

**Table 4:** Proportion of CPM based on the following TCM in MI-CPM prescriptions.

MI-CPMs	Rate
*Salvia miltiorrhiza-*based CPM	34.81%
*Ginkgo biloba-*based CPM	24.96%
*Panax notoginseng* based CPM	20.67%
*Gastrodia-*based CPM	7.15%
*Ligusticum Wallichii-*based CPM	4.90%
*Pueraria lobata-*based CPM	0.44%
*Carthamus tinctorius-*based CPM	0.43%
*Erigeron breviscapus-*based CPM	0.29%
*Crataegus pinnatifida-*based CPM	0.15%
Other TCM*-*based CPM	6.20%

*MI*-*CPMs* Chinese patent medicines with distinguishable main ingredients; Other TCMs included some Chinese herbal and animal medicines.

Among WM prescriptions, the top 10 most commonly used WMs were anti-hypertensives (32.82%), anti-thrombotic agents (16.06%), vasodilators (vasodilators except antihypertensives) (15.70%), anti-dementia agents (10.88%), lipid-lowering agents (9.58%), psychiatric/neurological agents (8.81%), anti-diabetics (5.03%), cerebral metabolism-enhancing drugs (0.33%), micro-circulation-improving drugs (0.27%), and others (0.52%) (Table 5).

**Table 5:** Proportion of each class of western medicine in WM prescriptions.

Western medicine	Rate
Anti-hypertensives	32.82%
Anti-thrombotic agents	16.06%
Vasodilators	15.70%
Anti-dementia agents	10.88%
Lipid-lowering agents	9.58%
Psychiatric/neurological agents	8.81%
Antidiabetics	5.03%
Cerebral metabolism-enhancing drugs	0.33%
Microcirculation-improving drugs	0.27%
Others	0.52%

Vasodilators were vasodilators except antihypertensive drugs, such as nitrates.

Others included medications for Parkinson’s disease (60.58%), epilepsy (23.34%), and so on.

**Figure 2:**
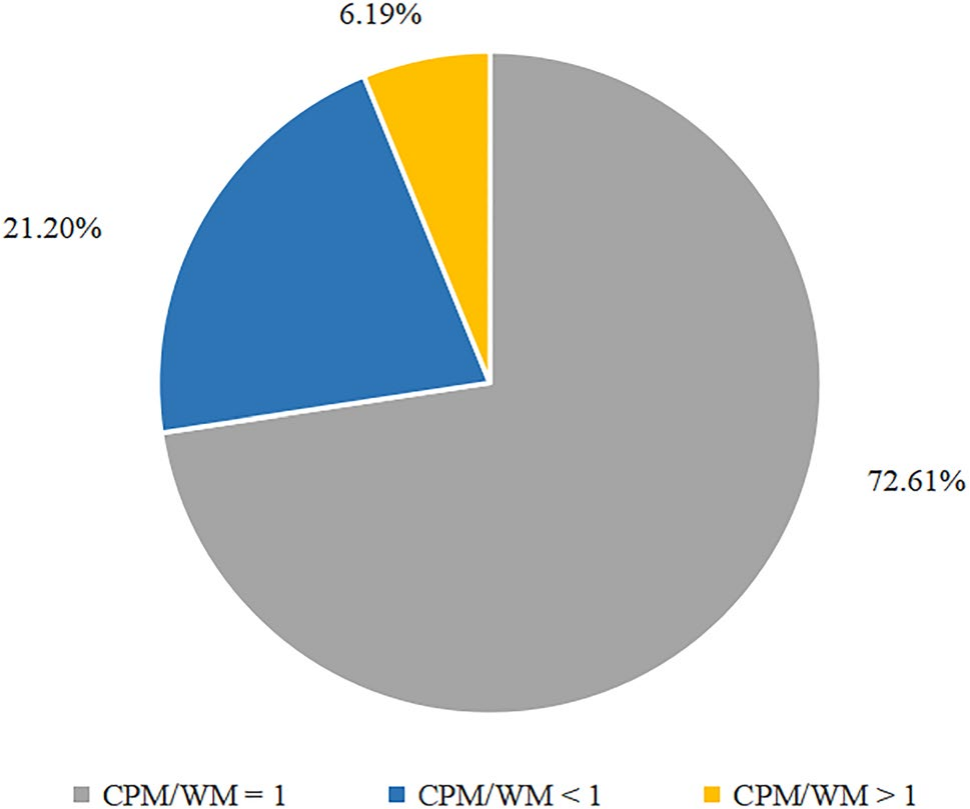
The proportion of prescriptions with different ratio of CPM and WM in CPM + WM prescriptions.

Among the CPM + WM prescriptions, 72.61% had a medication quantity ratio of 1:1 (CPM/WM = 1), 21.20% had a lower quantity of CPM compared to WM (CPM/WM < 1), and 6.19% had a higher quantity of CPM compared to WM (CPM/WM > 1) (Fig. 2).

Among the 1.98 million prescriptions analyzed, the top 10 most frequently prescribed medicines were: *Salvia miltiorrhiza*-based CPM (21.03%), anti-hypertensives (20.52%), *Ginkgo biloba*-based CPM (15.08%), *Panax notoginseng*-based CPM (12.49%), anti-thrombotic agents (10.04%), vasodilators (9.82%), anti-dementia agents (6.80%), lipid-lowering agents (5.99%), psychiatric/neurological agents (5.51%), and *Gastrodia*-based CPM (4.32%).

### Prescription pattern

**Figure 3:**
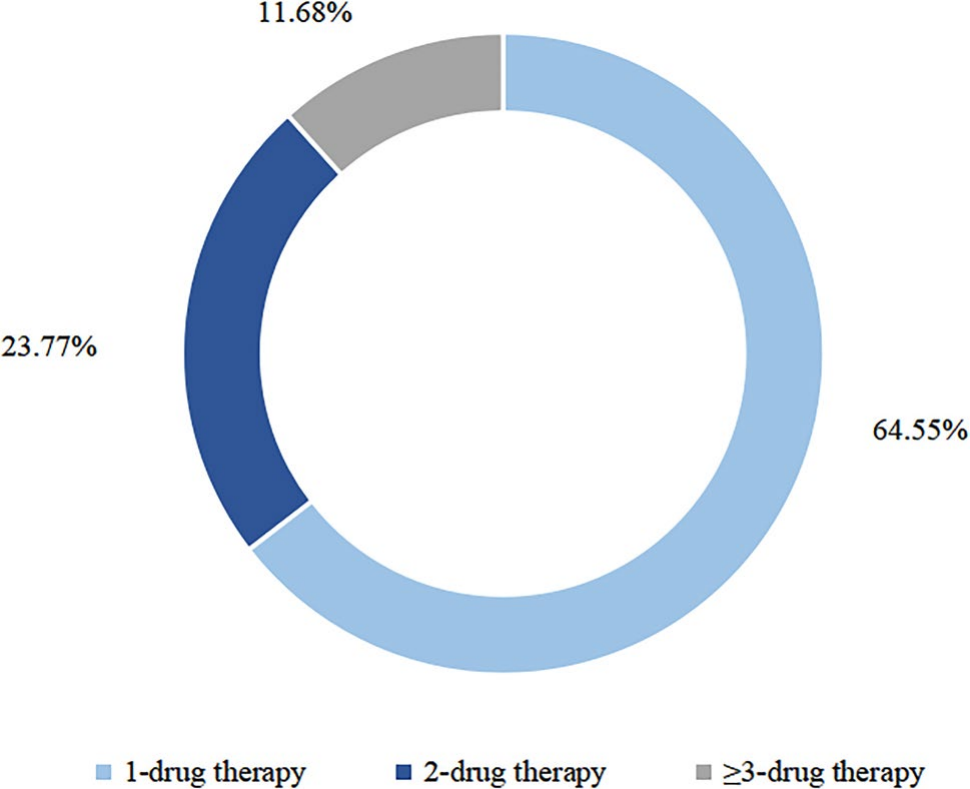
The proportion of 1-, 2-, and ≥ 3-drug prescriptions in the overall prescriptions.

All the prescriptions were divided into different types according to the number of drugs. The most common type was 1-drug regimen (64.55%), followed by 2-drug combination regimen (23.77%), and ≥ 3-drug combination regimen (11.68%) (Fig. 3).

#### Pattern of monotherapy regimens

Two forms of monotherapy were identified in the context of a 1-drug regimen: WM monotherapy and CPM monotherapy. The most frequently prescribed medications of WM monotherapy were anti-hypertensives (27.82%) and vasodilators (25.03%). The most frequently prescribed medications in CPM monotherapy were *Salvia miltiorrhiza*-based CPM (23.21%), *Ginkgo biloba*-based CPM (19.26%) and *Panax notoginseng*-based CPM (14.82%).

#### Pattern of combination regimens

**Figure 4:**
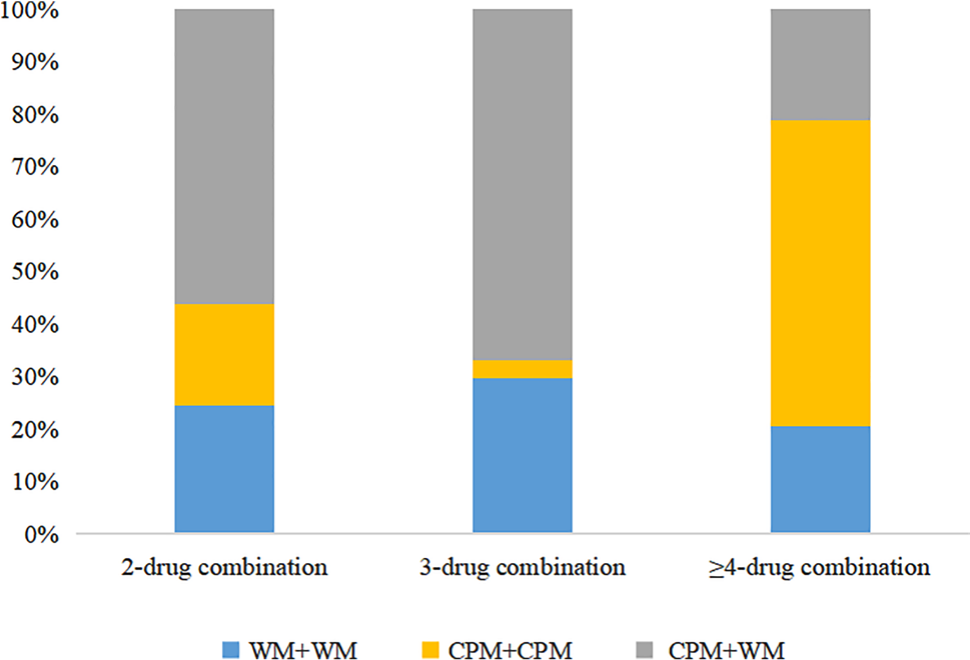
Proportion of CPM + WM, CPM + CPM, WM + WM in the combination therapies.

In the context of combination regimens, three primary forms were identified: WM + WM, CPM + WM, and CPM + CPM. Notably, CPM + WM emerged as the most common combination for both 2-drug and 3-drug combination regimens, representing 56.40% and 66.99% of prescriptions, respectively. Whereas CPM + CPM was the most commonly used in ≥ 4-drug combinations, accounting for 58.28% (Fig. 4).

Due to the small proportion of ≥ 4-drug combinations, no further analysis of specific drug combinations was conducted.

##### Pattern of CPM + WM prescriptions

The most commonly prescribed 2-drug combination regimen of CPM + WM were: *Salvia miltiorrhiza*-based CPM + anti-hypertensives (12.90%), *Ginkgo biloba*-based CPM + anti-hypertensives (10.33%) and *Panax notoginseng*-based CPM + anti-hypertensives (9.56%).

Within the 3-drug combination regimen, the most frequently prescribed combination was: *Salvia miltiorrhiza*-based CPM + antihypertensives + anti-thrombotic agents (5.67%).

##### Pattern of CPM + CPM prescriptions

The most commonly prescribed 2-drug combination regimen of CPM + CPM were: *Salvia miltiorrhiza*-based CPM + *Panax notoginseng*-based CPM (18.50%) and *Salvia miltiorrhiza*-based CPM + *Ginkgo biloba*-based CPM (11.70%).

Within the 3-drug combination regimen, the most frequently prescribed combinations was: *Salvia miltiorrhiza*-based CPM + *Panax notoginseng*-based CPM + *Ginkgo biloba*-based CPM (14.21%).

##### Pattern of WM + WM prescriptions

The most commonly prescribed 2-drug combination regimen of WM + WM were: anti-hypertensives + anti-thrombotic agents (13.76%), antihypertensives + antihypertensives (predominantly CCBs + ACEIs/ARBs/β-blockers) (13.56%), and antihypertensives + vasodilators (12.68%).

Within the 3-drug combination regimen, the most frequently prescribed combinations was: antihypertensives + anti-thrombotic agents + lipid-lowering agents (16.36%).

## Discussion

The study aimed to understand the drug prescription patterns of individuals with sequelae of CI by investigating prescriptions based on a real-world database and conducting big data analysis. The average age of all patients was 66.2 ± 11.4 years, with a slightly higher proportion of males (52.40%). These baseline findings are consistent with epidemiological studies on CI^12^. In addition, it is well established that hypertension is a major risk factor contributing to stroke DALYs^13^, and in this study, it emerged as the most common comorbidity associated with the sequelae of CI, accounting for 51.26% of cases.

CPM has been used for a long time as a common treatment in the management of the sequelae of CI, despite the lack of specific guideline recommendations. In this study, the CPMs that accounted for a higher percentage of statistical results were *Salvia miltiorrhiza*-based CPM, *Ginkgo biloba*-based CPM, and *Panax notoginseng*-based CPM. The efficacy of CPMs has been demonstrated in several clinical trials or basic research studies. For example, in a multicenter RCT, 2,200 patients with a history of ischemic stroke were randomized 1:1 to receive either Naoxintong capsule (*Salvia miltiorrhiza*-based CPM) or placebo in addition to standard care. Compared with the placebo group, the recurrence rate of ischemic stroke within 2 years was significantly lower in the Naoxintong group (P = 0.008), without increasing the risk of major bleeding in high-risk patients^14^. A systematic review and meta-analysis of 15 RCTs found that Ginkgo biloba extract (GBE, *Ginkgo biloba*-based CPM) improves neurological function and dependency in patients with different stages of ischemic stroke compared with conventional therapy and is generally safe for clinical use. The addition of GBE to conventional therapy improves Barthel Index scores and reduces neurological deficit scores in patients with acute ischemic stroke. In patients recovering from ischemic stroke (IS) or in the postictal period, GBE was superior to controls in improving dependency and neurological deficit scores^15^. An animal study shows that Xuesaitong (*Panax notoginseng*-based CPM) reduces brain infarct volume and alleviates neurological impairment 14 days after middle cerebral artery occlusion (MCAO), possibly by regulating microglial phenotypes via downregulation of the STAT3 signaling pathway^16^.

According to the theory of traditional Chinese medicine, patients in the sequelae stage of cerebral infarction often show the syndrome of qi deficiency and blood stasis, obstruction of meridians. Chinese patent medicines for promoting blood circulation, removing blood stasis and activating meridians can be appropriately selected^17^, such as *Salvia miltiorrhiza*-based CPM, *Panax notoginseng*-based CPM and *Ginkgo biloba*-based CPM.

Evidence-based practice guideline on integrative medicine for stroke^18^, focusing on the integrative approach of Western and Traditional Chinese Medicine (TCM) for stroke, is applicable across all levels of medical and rehabilitation institutions. It highlights the widespread use of this integrated approach in stroke prevention and treatment in China. Based on routine nursing and treatment, the addition of Chinese patent medicines has been shown to improve neurological damage in patients with IS. Initiating their use during the acute phase and continuing for 3–30 days may yield even greater benefits. Promising candidates include those CPMs made from *Salvia miltiorrhiza*, *Ginkgo biloba*, and *Panax notoginseng*. For patients with IS receiving internal medical treatment within 6 h of onset and without contraindications, in addition to intravenous thrombolysis, the immediate initiation of blood-activating and stasis-resolving TCM therapies can enhance neurological recovery. When combined with routine care and treatment after hemorrhage has been stabilized, TCM that activates blood and resolves stasis can also alleviate neurological deficits in patients with IS experiencing hemorrhagic transformation.

Although our study did not observe clinical effects or clinical outcomes, many previous studies have confirmed that CPM combined with WM can further improve the benefits of patients with CI or sequelae of CI. For example, in the treatment of CI, Xuesaitong (*Panax notoginseng*-based CPM) plus conventional treatment is conditionally recommended for the treatment of acute cerebral infarction to improve the total effective rate, National Institutes of Health Stroke Scale (NIHSS) score, China stroke scale (CSS) score, plasma viscosity (PV), and no serious adverse reactions were reported^19^. A systematic review and meta-analysis of 21 RCTs revealed that the combination of Salvianolate injection (*Salvia miltiorrhiza*-based CPM) with WM was associated with a higher overall response rate in the treatment of cerebral infarction compared to WM alone. Salvianolate injection demonstrated a positive impact on improving motor and cognitive functions without increasing adverse events^20^. In the treatment of CI sequela, when treating post-stroke epilepsy, a combination of oral Chinese herbal medicine and conventional WM outperformed WM monotherapy, reducing the duration of epileptic seizures and increasing the overall response rate^21^. Compared with standard WM treatment, TCM combined with WM treatment has a better effect on improving post-stroke depression (PSD)^22^. A meta-analysis of 1,367 patients with PSD from 18 RCTS showed that the combined treatment group exhibited improved scores on the Hamilton depression scale (HAMD), scores on the stroke scale (SSS), and enhanced Barthel index (BI) scores compared to the WM alone group. Additionally, gastrointestinal or neurological adverse events were also reduced in the combined treatment group^23^. A meta-analysis study of 8 RCTs involving 682 patients with neurological impairment after acute IS demonstrated that, compared with recombinant tissue-type plasminogen activator (rt-PA) of fibrinolysis monotherapy, patients treated with a combination of Salvianolic acid injection (*Salvia miltiorrhiza*-based CPM) and rt-PA may achieve better neurological recovery^24^. Collectively, these findings suggest that the integration of TCM with WM offers superior outcomes in the treatment of CI and its sequelae, without increasing adverse events.

Among the commonly prescribed MI-CPM, TCMs such as *Salvia miltiorrhiza, Ginkgo*, *Panax notoginseng*, *Gastrodia*, and *Ligusticum Wallichii* are frequently observed. These TCMs have a rich historical background in treating CI and have been extensively researched for their traditional medical principles and clinical efficacy.

*Salvia miltiorrhiza* possesses various pharmacological activities, including anti-atherosclerotic, anti-diabetic, anti-inflammatory, anti-oxidative and anti-tumor effects^25^. *Salvia miltiorrhiza* contains lipophilic constituents (tanshinone I, tanshinone IIa, tanshinone IIb, cryptotanshinone, dihydrotanshinone, etc.), as well as hydrophilic constituents (salvianolic acid A and B, danshensu, protocatechuic aldehyde, etc.)^26^. For example, tanshinone IIA exerts cardiovascular protection by reducing intimal thickening, inhibiting vascular smooth muscle cell proliferation and migration, and preventing thrombosis^27–29^. Cryptotanshinone (CPT) has shown anti-inflammatory and neuroprotective effects in mouse models of Alzheimer's disease (AD) by significantly reducing the expression of S100β, GFAP, COX-2, iNOS, and NFkBp65^30^. CPT also acts as an effective coronary dilator, improving microcirculation and increasing vascular blood flow^31^. *Salvia miltiorrhiza* hydrophilic extract has been found to significantly reduce the level of oxidized low-density lipoprotein (OX-LDL) in diabetes patients with coronary heart disease, potentially protecting against the development of diabetic cardiovascular disease^32^. *Salvia miltiorrhiza* injection antioxidant therapy has been shown to effectively reduce myocardial injury in children with congenital heart disease and reduce postoperative imbalance of vasoactive mediators^33^.

*Ginkgo biloba* has been found to potentially enhance memory and cognitive function in patients with Alzheimer's disease (AD) or Parkinson’s disease (PD). This is due to its pharmacological properties including anti-apoptotic, anti-oxidative, and anti-inflammatory effects^34^. *Ginkgo biloba* mainly contains flavonoids (such as quercetin, kaempferol, isorhamnetine) and several terpene trilactones (such as ginkgolides, bilobalide)^35^. Extract of *Gingko biloba* has shown significant neuroprotective effects against experimental focal cerebral ischemia^36^. Diterpene ginkgolides have been found to counteract astrocyte-mediated demyelination via the PAF-PAFR pathway^37^. Quercetin has a protective effect in the treatment of neurodegenerative and cerebrovascular diseases by inhibiting the NF-κB signaling pathway and regulating multiple kinase signaling cascades^38^. Animal studies proved that isorhamnetin can improve neurological function, enhance cognition and memory, and reduce the volume of cerebral infarction^39^. Clinical studies have demonstrated the efficacy of *Ginkgo biloba* extract containing ginkgolides A, B, and K, in improving neurological function in patients with IS^37^. Furthermore, quercetin supplements (500 mg/day) for 8 weeks have been found to significantly increase total antioxidant capacity in patients after myocardial infarction^40^. Diterpene ginkgolides meglumine injection has shown promising efficacy in improving functional outcome, neurological function and daily activities in patients with IS. It can be considered as an effective treatment option for elderly patients with IS^41^.

*Panax notoginse*ng has various pharmacological activities. It has been found to have beneficial effects on the cardiovascular and immune systems. The mechanism of actions include anti-atherosclerotic, hemostatic, anticoagulant, anti-oxidative, anti-inflammatory, and neuroprotective effects^42^. The main active components of *Panax notoginseng* are saponins, such as Ginsenoside Rg1, Ginsenoside Rb1, and Notoginsenoside R1. *Panax notoginseng* saponins (PNSs) have been shown to improve angiogenesis and microperfusion, as well as protect nerves^43,44^. They have demonstrated strong therapeutic effects on cardiovascular and cerebrovascular diseases, such as atherosclerosis, cerebral hemorrhage, and cerebral ischemic injury^44,45^. Ginsenoside Rb1 has been found to improve cognitive and sensorimotor deficits in stroke rats. This improvement is achieved by modulating the Akt/mTOR/PTEN signaling pathway and down-regulating caspase-3^46^. PNSs, including Ginsenoside Rg1 and Rb1, have also been shown to inhibit the apoptosis of hippocampal cells by scavenging ROS, increasing the expression of Trx-1, SOD-1 and HSP70, and restoring the anti-apoptotic Akt-NF-B signaling pathway^47^. Notoginsenoside R1 has been found to alleviate neuronal injury induced by β-amyloid through the inhibition of reactive oxygen species and regulating MAPK activation^48^. Study has shown that *Panax notoginseng* saponins significantly increase the probability of functional independence at 3 months in patients with IS, suggesting that this may be a safe and effective alternative treatment to improve the prognosis of this population^49^.

*Gastrodia* has various effects on the nervous system, cardiovascular system, and cerebrovascular system. It has been found to provide neuronal protection and regeneration, as well as treatment for neurodegenerative diseases. Additionally, it has sedative, hypnotic, analgesic, and anti-epileptic properties. In terms of the cardiovascular and cerebrovascular system, *Gastrodia* exhibits cardioprotective and cerebroprotective properties, along with the ability to effectively reduce blood pressure, lower blood sugar levels, and inhibit platelet aggregation^50^. The main active constituents of *Gastrodia* are gastrodin, its aglycone gastrodigenin, and polysaccharides^51,52^. Gastrodin exerts therapeutic effects on central nervous system diseases by regulating neurotransmitters, inhibiting microglia activation, up-regulating neurotrophic factors, and regulating mitochondrial cascade reactions^53^. In clinical practice, *Gastrodia* has been used to treat cognitive deficits and prevent neurodegenerative diseases, such as Alzheimer's disease (AD), Parkinson's disease (PD), and vascular dementia (VD)^54^. Clinical studies have shown that gastrodin effectively reduces the incidence of postoperative neurocognitive decline in patients undergoing cardiopulmonary bypass^55^, suggesting that gastrodin is a safe and effective in the prevention of neurocognitive decline.

The pharmacological effects of *Ligusticum Wallichii* primarily focus on the cardiovascular and cerebrovascular systems, providing neuroprotective, antioxidative, anti-inflammatory, anti-injury, and anti-tumor effects^56^. The bioactive components found in *Ligusticum Wallichii* can be categorized into four groups: phenols and organic acids (e.g. ferulic acid), phthalides (e.g. ligustilide, senkyunolide A), alkaloids (e.g. tetramethylpyrazine), and polysaccharides^56^. Administration of ligustilide effectively alleviates brain tissue ischemic damage through the Nrf2 and HSP70 signaling pathways^57^. *Ligusticum Wallichii* is clinically indicated for conditions related to the cardiovascular, cerebrovascular, nervous, and respiratory systems^58^. Clinical studies have shown that ferulic acid supplementation reduces total cholesterol, triglycerides, and HDL-C levels in patients with hyperlipidemia. It also significantly decreases oxidative stress biomarker malondialdehyde and inflammatory marker high sensitivity-C reactive protein (hs-CRP). These findings suggest that ferulic acid supplementation improves lipid profile, oxidative stress, OX-LDL cholesterol, and inflammation in patients with hyperlipidemia. Therefore, ferulic acid has the potential to reduce the risk factors associated with cardiovascular disease^59^.

Current studies have primarily concentrated on investigating the mechanisms by which TCMs promote blood circulation, dissolve blood stasis, regulate neural factors, suppress oxidative stress, alleviate inflammatory reactions, and facilitate neurological recovery. These effects are consistent with the underlying mechanisms responsible for functional impairments in sequelae of CI, and therefore demonstrate potential therapeutic effects.

In addition to CPMs, WMs were also used to treat the sequelae of CI in this study. The results imply that the WMs chosen by Internet doctors to treat sequelae of CI mainly play roles in two aspects. One category is drugs that can reduce risk factors, such as anti-hypertensives, lipid-lowering agents, and antidiabetics. The other category consists of drugs targeted at treating CI, including drugs that benefit blood vessels, mainly anti-thrombotic, vasodilators, and microcirculation-improving drugs, as well as drugs for treating and nourishing cerebral nerves, covering anti-dementia agents, psychiatric/neurological agents, and cerebral metabolism-enhancing drugs.

“2021 Guideline for the Prevention of Stroke in Patients With Stroke and Transient Ischemic Attack”^60^ suggests that symptomatic treatment should be given for cardiovascular-related comorbidities, and WMs reducing cerebral infarction-related risk factors (including hypertension, hyperlipidemia, and hyperglycaemia) are commonly used. For different disease etiology, such as large artery atherosclerosis, moyamoya disease, IS caused by small vessel disease, corresponding WMs treatment should be taken. The results of this study were generally consistent with the guideline recommendations.

There are some limitations in this study. First, patients can receive medical services in both offline and online hospitals. Due to the data permissions of different medical institutions and the diversity of diseases, we were unable to obtain comprehensive data on patients. Our research mainly focuses on online medical services. Second, during the online treatment process, the same patient may be treated by a different physician at each visit, and each physician may provide the patient with different medication choices based on his or her clinical experience and clinical guidelines. Our research mainly focuses on analyzing the usage trends of the same type of drugs. Third, limited to the existing internet hospital data, this study could not deeply confirm the efficacy and side effects of these online prescriptions, which should be differentiated when applied in clinical practice. Finally, it’s well known that some Chinese patent medicine have similar effects with some West medicine on the diseases. Therefore, the combined usage of Chinese patent medicine and West medicine might result in synergy effects to some extent. For example, ingredients such as notoginsenoside and tanshinone have a similar effect with calcium channel blockers, and thus need to be applied with great caution in combination. These were the limitations of this study, and the results were limited to online medication.

## Conclusion

In summary, this study conducted a comprehensive analysis of online prescription patterns for Chinese patients with sequelae of cerebral infarction. CPM played a significant role in the treatment of patients with sequelae of CI. MI-CPM, including *Salvia miltiorrhiza*, *Ginkgo biloba* and *Panax notoginseng* as the main ingredients, were the most frequently prescribed therapy for sequelae of CI. The combination of anti-hypertensives along with *Salvia miltiorrhiza*-based CPM emerged as the most prevalent combination therapy. This real-world study not only offered valuable insights into the prescription pattern in Chinese patients with sequelae of CI, but also served as a scientific reference for future clinical guidelines.

## Data Availability

All available data were included in the manuscript.

## Abbreviations

CI: Cerebral infarction
CPM: Chinese patent medicine
MI-CPM: CPM with distinguishable main ingredients
NI-CPM: CPM without distinguishable main ingredients
WM: Western medicine
DALYs: Disability-adjusted life years
TCM: Traditional Chinese medicine
AD: Alzheimer’s disease
GFAP: Glial fibrillary acidic protein
COX-2: Cyclooxygenase-2
iNOS: Inducible nitric oxide synthase
OX-LDL: Oxidized low-density lipoprotein
PD: Parkinson’s disease
PAF: Platelet-activating factor
PAFR: Platelet-activating factor receptor
IS: Ischemic stroke
NF-κB: Nuclear factor-kappa B
PNSs: Panax notoginseng saponins
ROS: Reactive oxygen species
SOD-1: Superoxide dismutase-1
Trx-1: Thioredoxin-1
HSP70: Heat shock protein 70
MAPK: Mitogen-activated protein kinase
VD: Vascular dementia
hs-CRP: High sensitivity-C reactive protein
HDL-C: High-density lipoprotein cholesterol
CCBs: Calcium channel blockers
ACEIs: Angiotensin-converting enzyme inhibitors
ARBs: Angiotensin receptor blockers
CPT: Cryptotanshinone
MCAO: Middle cerebral artery occlusion
RCT: Randomized controlled trial
rt-PA: Recombinant tissue-type plasminogen activator
NIHSS: National Institutes of Health Stroke Scale
CSS: China stroke scale
PV: Plasma viscosity
HAMD: Hamilton depression scale
SSS: Scores on the stroke scale
BI: Barthel index
